# Is normal body mass index a good indicator of metabolic health in Azar cohort population?

**DOI:** 10.15171/jcvtr.2019.09

**Published:** 2019-03-13

**Authors:** Mohammd Hossein Somi, Zeinab Nikniaz, Alireza Ostadrahimi, Amir Taher Eftekhar Sadat, Elnaz Faramarzi

**Affiliations:** ^1^Liver & Gastrointestinal Diseases Research Center, Tabriz University of Medical Sciences, Tabriz, Iran; ^2^Nutrition Research Center, Tabriz University of Medical Sciences, Tabriz, Iran; ^3^Consultant Anatomic and Clinical Pathologist, Pathology Department of Emam Reza Hospital, Tabrzi, Iran

**Keywords:** Metabolic Syndrome, Cardiometabolic Phenotypes, Obesity

## Abstract

***Introduction:*** Metabolic syndrome (Mets) has become most important public health problem in the world. We examined the association between Mets and different cardiometabolic phenotype in Azar cohort population.

***Methods:*** In the present study, the data of 13099 subjects who participated in Azar cohort study were cross-sectionally analyzed. Mets was defined according to the National Cholesterol Education Program’s Adult Treatment Panel III report (ATPIII) criteria. Participants were categorized into four cardiometabolic phenotypes including metabolically healthy Lean (MHL), metabolically unhealthy lean (MUHL), metabolically healthy Obese (MHO), metabolically unhealthy obese (MUHO) according to BMI cut–off point (25 kg/m2 ), and the presence of Mets.

***Results:*** Totally, the prevalence of Mets was 33.20% with the higher prevalence in women (40.1%). About 46.7% of participants were MHO and 1.6% of them were MHL. In both genders, MUHL had the highest prevalence of hyperglycemia, hypertrigliceridemia, hypo-HDL-cholestrolemia and Frahmingham 10-year CVD risk. In both MUHL and MUHO phenotypes, hypertriglyceridemia (OR: 31.97 [95% CI: 22.31, 45.81] and OR: 20.28 [95% CI: 17.32, 23.75]) and hypo-HDL cholestrolemia (OR:27.97 [95% CI: 17.35, 45.09] and OR:11.0 [95% CI: 9.62, 12.58]) are the strongest predictor of incidence of Mets. Also, the results of multinominal regression analyses indicated that in all cardiometabolic phenotypes, Framingham 10- year CVD risks had the lowest power for predicting of Mets incidence.

***Conclusion:*** Based on the results, in addition to obese individuals, multiple metabolic abnormalities were seen in normal weight individuals and these subjects are even at higher risk of developing Mets compared with metabolically obese individuals. So, it seems that decision on initiation of lifestyle interventions should not be only based on the BMI; rather metabolic status seems to be even more important.

## Introduction

Metabolic syndrome (Mets) includes a set of abnormalities including hypertension, dyslipidemia, impaired glucose tolerance, and abdominal obesity. These factors are correlated with increased risk of diabetes II, cardiovascular disease, cancer, and chronic renal failure which are the most common causes of hospitalization , morbidity, and early death.^1,2^

Mets has become a significant important public health problem in the world. Its prevalence is increasing in both developed and especially in developing countries.^3^ The prevalence of Mets varies between 16.3% and 33.4% in African and Asian countries.^4^ Further, the findings of Tehran Lipid and Glucose Study (TLGS) indicated that 33.7% of adult population of Tehran have Mets.^5^

In the recent decades, changes in lifestyle in developing countries and desire for westernization have led to rapid growth of Mets in these countries. Excess energy intake, sedentary lifestyle, and obesity have been known to be associated with Mets.

The results of most studies have documented overweight and obesity as the strongest predictors of Mets. However, among those who are identified as obese, some may not display any signs of typical metabolic disorders and have a lower risk of obesity-related complications.

Previously, it was reported that 10%-25% of obese individuals could be categorized as metabolically healthy obese (MHO).^6^ In this regard, Velho et al noted that prevalence of MHO ranged between 3.3 and 32.1% in men and between 11.4% and 43.3% in women.^7^ However, it does not mean that MHO subjects have harmless conditions. The findings of some studies suggested that they are at higher risk for developing hypertension, type 2 diabetes, and the metabolic syndrome compared to metabolically healthy subjects.^8,9^

On the other hand, a study on normal-weight adults (body mass index [BMI] <25.0 kg/m^2^) living in the United States showed that 24% of such adults were considered metabolically abnormal.^10^ This abnormality predisposes these groups of adult to chronic disease in comparison to metabolically healthy normal weight individuals. However, we are still not fully aware of the exact metabolic biomarkers that cause metabolically healthy individuals to become metabolically unhealthy during their life time. So, in the present study, the association between Mets and cardiometabolic phenotype was studied cross-sectionally in Azar cohort population.

## Materials and Methods

In the present study, the anthropometric, lipid profile, fasting blood sugar, and blood pressure levels were measured in 13099 subjects who participated in Azar cohort study. Azar cohort study is a part of a large Persian cohort study (The Prospective Epidemiological Research Studies of the Iranian Adults) launched in October 2014 and has been progressing up to now. This study has been explained in greater detail in previously published articles.^11,12^ Azar cohort was established in Shabestar in Eastern Azerbaijan province (North-west of Iran). Azar cohort has three phases including pilot, enrolment, and follow-up phases.

### Subjects

All eligible individuals with 35-70 years of age in Shabestar region were invited to participate in the study. Those included were inhabitants in Shabester for at least 9 months. The participants with severe psychiatric or physical illnesses and pregnant women were excluded from the study. The demographic information of the participants including age, gender, marital status, and education level was collected by a questionnaire.^11^

### Biochemical factors

Blood samples were collected after an overnight fast of 12 hours. Fasting blood sugar (FBS), serum triglyceride (TG), and high density lipoprotein (HDL) were determined by Pars Azmoon kits via enzymatic method.^11^

### Anthropometric measurements

Mounted tape was used for measuring the height to the nearest 1 mm, and Seca scale was used for recording the weight to the nearest 0.1 kg according to standard protocols. BMI was calculated via dividing weight (kg) by the square of height (m). The waist circumference (WC) was measured according to NIH guidelines. Women with WC ≥88 cm and men with WC of ≥102 cm were considered as abdominally obese.^13^ The frame size was calculated by height (cm)/wrist circumference (cm) ratio. Specifically, the frame size was classified as small, medium, and large.^14^

### Blood pressure measurements

The blood pressure was measured twice in each arm in the sitting position and according to Persian cohort protocol.^11^ There was a 2-minute rest between each two measurements. A person’s blood pressure was calculated as the average of the two measurements in each arm.

### Metabolic syndrome definition

We defined Mets according to the National Cholesterol Education Program’s Adult Treatment Panel III report (ATPIII) criteria.^15^ The subjects with three or more of the following conditions were defined as having Mets: WC ≥102 cm in men and ≥88 cm in women, TG ≥150 mg/dL (drug treatment for elevated TGs is an alternate indicator), HDL-C <40 mg/dL in men and <50 mg/dL in women; elevated blood pressure systolic ≥130 and/or diastolic ≥85 mm Hg (antihypertensive drug treatment in a patient with a history of hypertension is an alternate indicator); and elevated fasting glucose ≥100 (drug treatment of elevated glucose is an alternate indicator).

In this study, we categorized the participants into four cardiometabolic phenotypes according to BMI cut–off point (25 kg/m^2^) and the presence of Mets. Theses phenotypes included MHL (metabolically healthy lean Mets absent and BMI <25 kg/m^2^), MUHL (metabolically unhealthy lean Mets present and BMI <25 kg/m^2^), MHO (metabolically healthy obese Mets absent and BMI ≥25 kg/m^2^), and MUHO (metabolically unhealthy obese Mets present and BMI ≥25 kg/m^2^).

The Framingham risk of developing CVD was calculated based on age, HDL, total cholesterol level, systolic blood pressure, anti-hypertensive medication use, and current smoking status indicated as percentage.^16^ The subjects who had Framingham CVD risk score ≥10% were classified as high risk of developing CVD in 10 years.

### Statistical analysis

Statistical Package for the Social Sciences (SPSS, version 11.5, Chicago, IL) was used for the data analysis. Descriptive statistics were obtained for all study variables and reported as mean ± SD as well as number (percentage) where applicable. For comparing the baseline characteristics between women and men, the independent *t* test and χ2 test were used for quantitative and qualitative variables (education level, marital status) respectively. The multinomial logistic regression analysis was used for estimating crude and adjusted odds ratios (OR) and their corresponding 95% confidence intervals (95% CIs). Mets components (hypertension, high FBS, Hypo-HDL, cholesterolemia, hypertriglyceridemia, and abdominal obesity) and Framingham 10–year CVD risk ≥10% were considered as independent variables. Each variable was introduced in the model one by one. The effect of confounding factors (age, gender, educational level, marital status, current smoking status, and frame size) was adjusted and MHL was considered as the reference group. Statistical significance was considered as *P* value <0.05.

## Results

Table 1 presents the sociodemographic and anthropometric characteristics of the participants stratified by gender. Overall, 13099 (5821 men and 7278 women) were included in the analysis. The mean age of the participants was 49.52±9.27 years and their mean BMI was 28.83±4.91 kg/m^2^. About 14.9% of the participants were illiterate and 92.7% of them were married. There were significant differences between men and women in the mean of demographic and anthropometric values (*P* value < 0.001).

**Table 1:** Demographic and anthropometric characteristics of participants (n=13099)

	**Total (n=13099)**	**Males (n=5821)**	**Females (n=7278)**	***P*** ** value**
Age (y)	49.5±9.26	50 ±9.19	49.10±9.30	<0.001*
Weight (kg)	75.79±13.71	79.44±13.63	72.89±13.07	<0.001*
Height (cm)	162.24±9.61	170.12±6.68	155.94±6.41	<0.001*
BMI (kg/m^2^)	28.83±4.91	27.42±4.33	29.96±5.06	<0.001*
Education level				<0.001**
Illiterate	1956(14.9)	415(7.1)	1541(21.2)	
≤High school/diploma	9925(75.8)	4647(79.8)	5278(72.5)	
≥ College degree	1203(9.2)	753(12.9)	450(6.2)	
Marital status				<0.001**
Single	198(1.5)	35(0.6)	163(2.2)	
Married	12144(92.7)	5750(98.8)	6394(87.9)	
Widowed	660(5.1)	16(0.3)	644(8.9)	
Divorced	94(0.7)	19(0.3)	75(1)	

Abbreviation: BMI, Body mass index * Independent *t* test; ** χ2 test.

The mean of Mets components and the percentage of cardiometabolic phenotypes are reported in Table 2. Totally, the prevalence of Mets was 33.20% with a higher prevalence in women (40.1%). About 46.7% of the participants were metabolically healthy obese and 1.6% of them were metabolically unhealthy lean. There were significant differences between men and women in the mean of Mets components values (*P* value <0. 01), except for diastolic blood pressure (*P* value = 0.60).

**Table 2:** Metabolic syndrome components characteristics of participants

**Variable**	**Total**	**Males**	**Females**	***P*** ** value**
	**Mean ± SD**	**Mean ± SD**	**Mean ± SD**	
Waist circumference(cm)	94.41±11.30	95.54±11.20	93.51±11.3	<0.001
Systolic blood pressure (mm Hg)	114.58±17.36	113.65±16.93	115.33±17.65	<0.001
Diastolic blood pressure (mm Hg)	73.74±9.66	73.69±9.47	73.78±9.82	0.60
Triglyceride(mg/dL)	149.12±84.25	155.81±93.80	143.77±75.33	<0.001
High density lipoprotein (mg/dL)	98.43±32.68	41.81±9.31	49.04±10.88	<0.001
Fasting blood sugar (mg/dL)	45.83±10.82	97.58±30.5	99.11±34.31	0.008
	**No. (%)**	**No. (%)**	**No. (%)**	
Metabolic syndrome	4353(33.2)	1436(24.7)	2917(40.1)	<0.001
MHL	2613(19.9)	1611(27.7)	1002(13.8)	<0.001
MUHL	204(1.6)	87(1.5)	117(1.6)	
MHO	6122(46.7)	2770(47.6)	3352(46.1)	
MUHO	4149(31.7)	1349(23.2)	2800(38.5)	
Framingham 10–year CVD risk ≥ 10 %	5089(38.9)	2652(45.6)	2437(33.5)	<0.001

MHL metabolically healthy lean (MetS absent and BMI < 25 kg/m^2^), MUHL metabolically unhealthy lean (MetS present and BMI <25 kg/m^2^),MHO metabolically healthy obese (MetS absent and BMI ≥25 kg/m^2^), MUHO metabolically unhealthy obese (MetS present and BMI ≥ 25 kg/m^2^).

The prevalence of abnormal metabolic status stratified by cardiometabolic phenotypes is presented in Table 3. In both genders, the prevalence of hyperglycemia, hypertriglyceridemia, and hypo-HDL-cholesterolemia was the highest in the MUHL group. However, the prevalence of hypertension and abdominal obesity was the highest in the MUHO group. Finally, the highest prevalence of Framingham CVD rsik ≥10% was observed in the MUHL phenotype (78.2% in men; 65% in women).

**Table 3:** The prevalence of abnormal metabolic status stratified by cardiometabolic phenotype and sex

**Variables**	**Male**				***P*** *	**Female**				***P*** *
	**MHL** ** (n=1611)**	**MUHL** ** (n=87)**	**MHO** ** (n=2770)**	**MUHO** ** (n=1349)**		**MHL** ** (n=1002)**	**MUHL** ** (n=117)**	**MHO** ** (n=3352)**	**MUHO** ** (n=2800)**	
Hyperglycemia	203 (12.6)	70 (80.5)	433 (15.6)	822 (60.9)	<0.001	141 (14. 1)	78 (66.7)	310 (9.2)	1552 (55.4)	<0.001
Hypertriglyceredmial	271 (16.8)	77 (88.5)	820 (29.6)	1027 (76.1)	<0.001	107 (10.7)	80 (68.4)	372 (11.1)	1788 (63.9)	<0.001
Hypo-HDL cholestrolemia	454 (28.2)	80 (92)	953 (34.4)	1062 (78.7)	<0.001	397 (39.6)	104 (88.9)	1295 (38.6)	2259 (80.7)	<0.001
Abdominal obesity	1 (0.1)	1 (1.1)	631 (22.8)	966 (71.6)	<0.001	54 (5.4)	53 (45.3)	2250 (67.1)	2710 (96.8)	<0.001
Hypertension	181 (11.2)	49 (56.3)	454 (16.4)	800 (59.3)	<0.001	134 (13.4)	78 (66.7)	528 (15.8)	1725 (61.5)	<0.001
Frame size	
Small	195 (12.1)	6 (6.9)	11 (0.4)	1 (0.1)	<0.001	55 (5.5)	0 (0)	3 (0.1)	3 (0.1)	<0.001
Medium	809 (50.2)	32 (36.8)	353 (12.7)	114 (8.5)	<0.001	341 (34)	32 (27.4)	174 (5.2)	58 (2.1)	<0.001
Large	607 (37.7)	49 (56.3)	2406 (86.9)	1234 (91.5)	<0.001	606 (60.5)	85 (72.6)	3175 (94.7)	2739 (97.8)	<0.001
Metabolic components										
0	784 (48.7)	-	611 (22.1)	-		390 (38.9)	-	377 (11.2)	-	<0.001
1	544 (33.8)	-	1027 (37.1)	-		391 (39)	-	1195 (35.7)	-	<0.001
2	283 (17.6)	-	1132 (40.9)	-		22 (22.1)	-	1780 (53.1)	-	<0.001
3	-	71 (81.6)	-	823 (61)	<0.001	-	80 (68..4)	-	1526 (54.5)	<0.001
4	-	16 (18.4)	-	422 (31.3)	<0.001	-	32 (27.4)	-	914 (32.6)	<0.001
5	-	-	-	104 (7.7)	-	-	5 (4.3)	-	360 (12.9)	<0.001
Framingham 10–year CVD risk ≥ 10 %	683 (42.4)	68 (78.2)	1076 (38.9)	823 (61)	<0.001	216 (21.6)	76 (65)	639 (19)	1505 (53.8)	<0.001

Mets: metabolic syndrome; MHL: metabolically healthy lean (MetS absent and BMI < 25 kg/m^2^); MUHL; metabolically unhealthy lean (MetS present and BMI < 25 kg/m^2^); MHO: metabolically healthy obese (MetS absent and BMI ≥25 kg/m^2^); MUHO: metabolically unhealthy obese (Mets present and BMI ≥ 25 kg/m^2^); High FBS: FBS ≥100 mg/dL (drug treatment of elevated glucose); Hypertriglycerdemia: TG ≥ 150 mg/dL; Hypo-HDL cholestrolemia: Male <40 mg/dL, Female <50 mg/dL; Abdominal obesity: Male WC ≥102 cm, female WC ≥88 cm; Hypertension: Systolic ≥ 130 and/or diastolic 85 mm Hg (antihypertensive drug treatment in a patient with a history of hypertension). The values are in No. (%). * *P* value of chi-squre.

Considering the results from logistic regression analyses (Table 4), compared to MHL phenotype (reference group), MUHL and MUHO phenotypes had higher odds of being associated with hypertension, hyperglycemia, hypertriglyceridemia, abdominal obesity, hypo-HDL, cholesterolemia, and Frahmingham 10-year CVD risk in both unadjusted and adjusted models. However, in terms of MHO, compared with MHL phenotype, this phenotype had significantly greater odds of association only with hypertension, hypertriglyceridemia, abdominal obesity, and hypo-HDL cholesterolemia.

**Table 4:** Predictor factors of metabolic syndrome

**Variables**	**MUHL**				**MHO**				**MUHO**			
	**OR** ^a^ ** (95%CI)**	***P ***	**OR** ^b^ **(95%CI)**	***P***	**OR** ^a^ ** (95%CI)**	***P***	**OR** ^b^ ** (95%CI)**	***P***	**OR** ^a^ ** (95%CI)**	***P***	**OR** ^b^ ** (95%CI)**	***P***
Hypertension	12.03 (8.85-16.35)	<0.001	8.76 (6.30-12.18)	<0.001	1.39 (1.18-1.62)	<0.001	1.39(1.18-1.62)	<0.001	11.34 (9.92-12.95)	<0.001	10.04(8.58-11.74)	<0.001
Hyperglycemia	17.43 (12.56-24.19)	<0.001	15.09 (10.07-21.12)	<0.001	0.91 (0.79-1.04)	0.18	0.91 (0.78-1.06)	0.22	8.82 (7.75-10.03)	<0.001	8.25(7.1-9.60)	<0.001
Hypertriglyceridemia	19.75 (14-27.85)	<0.001	31.97 (22.31-45.81)	<0.001	1.43 (1.26-1.62)	<0.001	1.63 (1.40-1.89)	<0.001	12.47(10.98-14.16)	<0.001	20.28(17.32-23.75)	<0.001
Abdominal obesity	16.74 (11.11-25.22)	<0.001	11.16 (7.18-17.33)	<0.001	41.34 (31.5-54.215)	<0.001	39.20(29.53-52.03)	<0.001	-	-	-	**-**
Hypo-HDL cholestrolemia	19.04 (11.92-30.43)	<0.001	27.97 (17.35-45.09)	<0.001	1.20 (1.09-1.32)	<0.001	1.16(1.03-1.29)	<0.001	8.3(7.42-9.28)	<0.001	11.0(9.62-12.58)	<0.001
Framingham 10–year CVD risk ≥ 10 %	4.78 (3.47-6.58)	<0.001	4.30 (2.67-6.92)	<0.001	0.73 (0.66-0.81)	<0.001	1.07(0.89-1.27)	0.47	2.5(2.65-2.77)	<0.001	4.26(3.56-5.11)	<0.001

^a^ Unadjusted OR: odds ratio; ^b^ Adjusted for age, gender, smoking, education level, marital status, frame size. MUHL; metabolically unhealthy lean (MetS present and BMI < 25 kg/m^2^); MHO: metabolically healthy obese (MetS absent and BMI ≥25 kg/m^2^); MUHO: metabolically unhealthy obese (MetS present and BMI ≥ 25 kg/m^2^) Hypertension: Systolic ≥ 130 and/or diastolic 85 mm Hg (antihypertensive drug treatment in patient with a history of hypertension); High FBS: FBS ≥100 mg/dL (drug treatment of elevated glucose) ;hypertriglycerdemia:TG ≥150 mg/dL; Hypo-HDL cholestrolemia: Male <40 mg/dL, female <50 mg/dL; Abdominal obesity: Male ≥102 cm, female ≥88 cm.

In both MUHL and MUHO phenotypes, hypertriglyceridemia (OR:31.97 [95% CI: 22.31, 45.81] and OR:20.28 [95% CI: 17.32,23.75]) and hypo-HDL cholesterolemia (OR:27.97 [95% CI: 17.35, 45.09] and OR:11.0 [95% CI: 9.62, 12.58]) were the strongest predictors of incidence of Mets. On the other hand, the results of multinomial regression analyses indicated that in all cardiometabolic phenotypes, Framingham 10-year CVD risks had the lowest power for predicting of Mets incidence.

## Discussion

The cross-sectional analysis of the Azar cohort data revealed that the prevalence of the Mets in our population was 33.2% which was more prevalent in women compared to men. The prevalence of Mets in our population was approximately the same as previous reports from Tehran Lipid and glucose study (33.7%)^5^ as well as other developing countries such as Turkey (36.6%)^17^ along with developed countries such as the United States (34.7%).^18^ However, it was higher than the prevalence of Mets in Brazil (29%)^19^ and Europe (24%).^20^ According to the results of the present study, the Mets prevalence was less in men than in women. The same results were found in a previous study from Iran.^21^ Some factors including use of hormonal contraceptives, pregnancy, lactation, and menopause predispose women to develop Mets.^22^

In the present study, the prevalence of MHL, MUHL, MHO and MUHO was 19.9%, 1.6%, 46.7%, and 31.7% respectively. The prevalence of MHO in our study (47.6% for men and 46.1% for women) was higher than in the previous studies which have reported it as 2.3%-19% for men and 7.3%-28.4% for women.^23^ This observed discrepancy may be due to use of different cut-off points for defining MHO phenotype in different studies. As mentioned above, in the present study, BMI ≥25 kg/m^2^ was used for classification of cardiometabolicphenotypes while the previous studies used BMI cut-off ≥30 kg/m^2^.

Considering the findings of the present study, MUHL and MUHO phenotypes had the maximum odds for Mets components such as hypertriglyceridemia and hypo-HDL cholesterolemia, respectively. Interestingly, MUHL phenotype had the highest prevalence of metabolic abnormalities and Framingham 10-year CVD risk ≥10 year among the cardiometabloic phenotypes. These findings are consistent with Aung et al’s^24^ study who noted that the mean systolic blood pressure and TG of MUH-NW individuals was higher than that of MHO individuals. The existing reports also suggested that MUHL phenotype was at increased risk of cardiovascular disease, type 2 diabetes, and mortality,^25-27^, and our results are in line with the previous findings. Rhee et al^28^ in the Korean population showed that metabolically unhealthy participants had a significantly higher risk for development of type 2 diabetes compared with metabolically healthy participants, regardless of their BMI status. In another study in the United States, individuals with MUHL phenotype were at higher risk of developing CVD and diabetes.^24^ These results may be due to the effect of visceral obesity on deterioration of metabolic health, as fat distribution plays an important role in developing metabolic disorder and increasing chronic diseases. The relationship between fat distribution and metabolic disorders is complicated. It has been shown that visceral obesity and ectopic fat deposit can increase the risk of insulin resistance and inflammatory factors.^29-31^ In this regard, compared with MHL phenotype, MUHL individuals have shown reduced compensatory insulin response.^32^ Previous studies have reported that physical activity increased the likelihood of promoting metabolic health status.^33^ Further, MUHL subjects proved to have higher levels of hs-CRP, where this condition is significantly associated with high levels of blood sugar and lipids.^28^

It has been reported that people with MHO phenotype are not at increased risk for hyperglycemia and hyperlipidemia.^34-37^ The results of the present study showed that individuals with MHO phenotype were at increased risk of abnormal lipid profile and hypertension, which is in contrast to the aforementioned studies. These findings were in line with the result of study conducted on the data of NHANES III study and suggested that individuals with metabolically healthy obese phenotype were at increased of morbidity and mortality.^26^ Additionally, the recent studies suggest that MHO phenotype frequently progresses to MUHO phenotype.^38,39^ Currently, it is not obvious why MHO individuals are less likely to develop cardiometabolic risk factor compared to MUHL, but it has been suggested that this may be due to the higher fitness level of these individuals.^37^

The strength of the present study included using a large population-based sample, duplicate measuring of anthropometric indices, the blood pressure, and determining the frame size of the participants. However, considering insufficient covariates included in the present study, these results should be interpreted with caution. Other covariate rather than included ones may influence the association between cardiometabolic phenotypes and Mets components such as dietary factors and physical activity pattern. The physical fitness and also percentage of body fat were not measured either. The low number of MUHL subjects in different subgroups was another limitation of this study which may lead to a wide range of confidence interval in multinomial regression. Moreover, because of the cross-sectional analysis of the data, causal relationships may not be established.^37^

In conclusion, based on the results, in addition to obese individuals, multiple metabolic abnormalities were seen in normal weight individuals and these subjects are even at higher risk of developing Mets compared with metabolically obese individuals. So, it seems that decision on initiation of lifestyle interventions should not be only based on the BMI; rather metabolic status seems to be even more important.

## Ethical approval

All participants signed a written informed consent and it was approved by the Ethics Committee of Tabriz University of Medical Sciences (tbzmed.rec.1393.205).

## Competing interests

The authors declared no potential conflicts of interest with respect to the research, authorship, and/or publication of this article.

## Funding

This research was supported by the Liver and Gastrointestinal Disease Research Center, Tabriz University of Medical Sciences.

## Acknowledgment

The authors also are deeply indebted to all participants. We appreciate the contribution by investigators and staff of the Azar Cohort Study. We thank the close collaboration of the Shabestar Health center. Also, we would like to thank the Persian Cohort study staff for their technical support.
